# Phenotypic Screening in *C. elegans* as a Tool for the Discovery of New Geroprotective Drugs

**DOI:** 10.3390/ph13080164

**Published:** 2020-07-25

**Authors:** Sven Bulterijs, Bart P. Braeckman

**Affiliations:** Laboratory of Aging Physiology and Molecular Evolution, Department of Biology, Ghent University, 9000 Ghent, Belgium

**Keywords:** drug screening, phenotypic screen, target-based screen, geroprotective drug, aging, longevity

## Abstract

Population aging is one of the largest challenges of the 21st century. As more people live to advanced ages, the prevalence of age-related diseases and disabilities will increase placing an ever larger burden on our healthcare system. A potential solution to this conundrum is to develop treatments that prevent, delay or reduce the severity of age-related diseases by decreasing the rate of the aging process. This ambition has been accomplished in model organisms through dietary, genetic and pharmacological interventions. The pharmacological approaches hold the greatest opportunity for successful translation to the clinic. The discovery of such pharmacological interventions in aging requires high-throughput screening strategies. However, the majority of screens performed for geroprotective drugs in *C. elegans* so far are rather low throughput. Therefore, the development of high-throughput screening strategies is of utmost importance.

## 1. Introduction

Over the last century, improvements in diet, living conditions, education, clean drinking water, development of vaccines and medical care have strongly reduced the mortality rate from infectious disease, particularly in early life [1,2]. Towards the final decades of the 20th century, medical progress started to tackle the diseases of aging, particularly heart disease, leading to a further increase in life expectancy [3]. These developments have resulted in a progressive linear increase in life expectancy at birth from 48.9 years in 1900 to 79.0 years in 1995 for females born in the US [4,5,6]. As a consequence, the number of elderly people has increased rapidly. At the turn of the 20th century, only 4.1% of the US population was 65 or older. By 2014, this number had increased to 14.5%, and it is predicted to further increase to 22.1% by the middle of this century [7].

The aging process leads to a progressive loss of function and a decline in resilience of the organism [8,9]. This in turn increases the susceptibility to a wide diversity of chronic diseases such as coronary artery disease, stroke, senile systemic amyloidosis, Alzheimer’s disease, osteoporosis, sarcopenia and various cancers [10,11,12,13]. Furthermore, the age-related decline in immune function, known as immunosenescence, increases the susceptibility to communicable diseases [14]. Consequently, aging is the prime cause of disease, disability and death worldwide [15,16]. In recent decades, many interventions have been found to increase life- and healthspan in a variety of model organisms such as the nematode *Caenorhabditis elegans*, *Drosophila melanogaster*, mice and rats. The first intervention discovered to increase life- and healthspan was dietary restriction, the act of reducing total calorie intake or the intake of specific nutrients without malnutrition [17,18,19]. This was followed by pharmacological and genetic interventions [20,21,22,23]. For example, reducing insulin/insulin-like growth factor-1 (IGF-1) signaling resulted in a robust lifespan extension in *C. elegans*, *Drosophila* and mice [24,25]. These success stories have led to the formulation of the geroscience hypothesis: interventions that slow down aging will simultaneously prevent, delay and/or reduce the severity of many age-related diseases [16,26,27]. As dietary interventions prove to be challenging for most people to adhere to in the long term [28,29] and genetic interventions still suffer from many technical, ethical and safety problems [30,31], the main potential in the near future for human clinical translation is the development of pharmacological interventions in the aging process: so-called geroprotective drugs [22,32,33,34]. Multiple drugs such as rapamycin, metformin, spermidine, senolytics, lithium and acarbose have been found to extend lifespan in model organisms [21,22,32,35]. In fact, the first randomized, controlled clinical trial, the TAME (Targeting Aging with Metformin) trial, has recently cleared the last obstacle, securing enough funding, and so the trial should start in the near future [36]. What makes this trial unique is that it specifically aims to evaluate the effects of the drug intervention on the aging process through the use of a composite outcome that includes cardiovascular events, cancer, dementia and mortality as a primary endpoint [37].

## 2. Target-Based Versus Phenotypic Screening

### 2.1. The Pros and Cons of Target-Based Versus Phenotypic Screening

While early drug discovery was mostly a serendipitous affair based on observations over centuries that certain substances had healing properties in specific diseases, drug discovery became more scientific at the beginning of the 20th century [38]. Paul Ehrlich and colleagues synthesized 900 compounds and systematically screened them in syphilis-infected rabbits for their effectiveness leading to the discovery of compound 606, which became known as Salvarsan. Salvarsan was the first man-made antibiotic and came into general use in 1910 [39,40,41]. Currently, the two main paradigms in the field of drug discovery are phenotypic screens and target-based screens (Table 1). Other methods such as fragment-based screens and virtual screens exist to identify chemical matter that likely binds to a functional site within a protein of interest but will not be covered in this review. Target-based screens use isolated targets (such as proteins) and screen for compounds that inhibit or activate the target by employing a biochemical readout (for example inhibition of catalytic activity of the target). In contrast, phenotypic screens employ cell culture, tissues or whole organisms and use a phenotype, such as death, as a readout. Before the advent of recombinant gene technology, most drug screens employed phenotypic screening. For example, most antibiotics were discovered by screening compounds for their ability to kill or inhibit the growth of bacterial cells [42]. Beta-blockers were developed by ex vivo studies in heart tissue from guinea pigs [43,44]. Various heat, pressure, electric shock or noxious stimulus assays in experimental animals have been used for the identification of analgesics [45].

The major limitation of phenotypic screens is that the mechanism of action of drug hits is unknown and that significant resources might have to be spent in unraveling the mechanism. In fact, it has been argued that the difficulty of target identification is the main reason phenotypic screens are not more widely used in the pharmaceutical industry [46]. Although knowing the mechanism of action is preferable, it is not an absolute requirement for regulatory approval [47]. Indeed, according to one study, 7% of known drugs lack a molecular mechanism of action [48]. Furthermore, the throughput of phenotypic screens has historically been rather limited, reducing the number of compounds that can be screened.

The advent of ever larger compound libraries has necessitated an increase in throughput, and this eventually gave rise to high throughput screening (HTS) in the 1980s. This trend was further catalyzed by improvements in: (i) genetic research leading to a rapid expansion in the number of new drug targets; (ii) recombinant DNA technology allowing the production of purified recombinant proteins for biochemical assays; (iii) assay miniaturization; (iv) the development of liquid handling technologies and robotic automation; and (v) advancements in data processing [47,49]. With these advances, some companies have started to conduct upwards of 100,000 assays a day, which has been referred to as ultra-high throughput screens [49,50]. HTS can either be cell-based or ex vivo biochemical target-based screens [51].

In a landmark paper, Swinney and Anthony (2011) analyzed data on FDA drug approvals between 1999 and 2008 and found that phenotypic screens are more likely to lead to the discovery of first-in-class drugs compared to target-based screening [52]. However, in contrast, another study that looked at the drug approvals by the FDA between 1999 and 2013 found that target-based screens were responsible for the majority of first-in-class drugs during that period [53]. The limitations of pure target-based screening approaches for drug discovery were highlighted by a massive study conducted by researchers from GlaxoSmithKline who looked for new antibacterial drugs by running HTS screens against 70 targets. This effort took seven years to complete and resulted in only five leads, none of which could be progressed further [54]. Many targets identified in the literature are insufficiently validated, and this may contribute to the large attrition rates of drug leads during animal testing or early clinical trials [55,56,57]. The target agnostic nature of phenotypic screens can also be considered a major strength as it allows for the discovery of truly new therapeutic targets. In target-based screening, a drug is optimized for its interaction with a single target, while it is now appreciated that some very successful drugs work by being promiscuous for multiple targets that are together involved in driving disease pathophysiology [58,59,60,61].

These new insights have led to a revival of phenotypic screening in the last decade [47,62]. While earlier phenotypic screens used either cells or mammals (either screening in vivo or on isolated tissues), in more recent times, small model organisms such as *C. elegans*, *Drosophila melanogaster*, zebrafish and *Xenopus laevis* have been introduced for phenotypic drug screening [63,64]. These organisms are smaller and cheaper compared to the use of mammals in phenotypic screening campaigns. In addition, the use of invertebrates such as *C. elegans* and *Drosophila* raise no ethical concerns. Compared to cell-based phenotypic screening, these small model organisms allow for drug screening in the context of cell–cell and cell–extracellular matrix interactions under physiological conditions [65]. Whole-animal screens provide information about important pharmacokinetic, pharmacodynamic, toxicological and off-target activity of the screened compounds at an early stage thereby possibly reducing attrition rates during downstream phases of the drug development process. As each phase in drug development is more costly than the previous one, the mantra in the pharmaceutical industry is to “fail fast, fail cheap” [66]. Furthermore, these small model organisms offer the advantage that more complex phenotypes (even behavioral assays) can be used as read-outs. Therefore, phenotypic screens in whole organisms are particularly well suited for screens looking at ill understood, complex or multisystem diseases such as neurological disorders and aging. Hence, small model organisms represent the middle ground between cell-based and mammalian phenotypic screens. 

### 2.2. Phenotypic Screening for Geroprotective Compounds

To improve the translational potential of phenotypic drug screening three criteria for phenotypic assays were proposed: assay system, stimulus and readout [56]. The first criterion deals with the disease-relevance of the used assay system. Many phenotypic screens have used cell lines that possess genetic abnormalities or differ from the cell types in which the disease normally manifests itself hence limiting the physiological relevance of the hits obtained from the screen. For example, a group of drugs known as correctors for their ability to suppress defects in the intracellular trafficking of the chloride channel CFTR induced by the F508del mutation, the most common cause of cystic fibrosis, was screened and it was found that the majority of drugs were only active in one of the two tested cell lines. Furthermore, of the nine drugs that were active in both cell lines, only two corrected the defect in primary human bronchial epithelial cells [67]. The second criterion for phenotypic screens is the stimulus employed to evoke the disease phenotype of interest. This can be omitted by using assay systems that intrinsically contain the stimulus, such as patient derived primary cells that contain the disease-causing mutation. We briefly discuss the use of stress assays in the discovery of geroprotective drugs for which the choice of an appropriate stimulus is essential in Section 5.3. For example, in *C. elegans*, heat stress resistance is a better predictor of lifespan than oxidative stress resistance [68]. This is likely due to the fact that heat stress resistance is a measure for proteome stability, a well-known determinant of longevity [69,70,71], while the role of oxidative stress in aging, particularly in *C. elegans*, has been questioned [72]. Finally, the assay readout is also imperative. Currently, there is a lack of validated readouts for cellular aging that are predictive of organismal health or longevity [73]. Therefore, most phenotypic screens that look for geroprotective drugs use small model organisms and employ lifespan as the readout. Phenotypes used in these screens should be well defined and lifespan is a quantifiable phenotype for which appropriate statistics have been developed [46]. As aging is a systemic process that leads to the deterioration of multiple tissues, organs and physiological functions, it is better represented by phenotypic screens in small model organisms compared to cell-based screens [74]. However, cell-based phenotypic screens for geroprotective drugs have been carried out in the past. For example, primary murine embryonic fibroblasts from DNA repair deficient *Ercc1^-/-^* mice that are very sensitive to cellular senescence when grown in atmospheric oxygen have been used to screen for drugs that either kill senescent cells (senolytic drugs) or drugs that suppress senescent phenotypes (senomorphic drugs). Cellular senescence was measured through the detection of senescence-associated β-galactosidase activity with a fluorescent substrate and quantified with the IN Cell Analyzer 6000 leading to the identification of HSP90 inhibitors as a novel class of senolytic drugs [75]. Similar cell-based screens have been carried out, leading to the identification of KU-60019 [76], cardiac glycosides [77] and fenofibrate [78] as senolytic drugs. Recently, over 100,000 small molecules were screened for their ability to increase oxidative stress resistance in primary human lung fibroblasts. This effort resulted in the discovery of 209 primary hits. After several follow-up assays, the list was reduced to a set of 32 core hits that were tested for their effect on lifespan in *C. elegans*, resulting in the identification of nine compounds that increased lifespan by around 10–50%. However, seven compounds were found to have toxicity issues and one compound fit the criteria for “pan assay interference compounds” (PAINS). Hence, the screen resulted in the identification of one prime candidate, the chalcone Gr-4D, for follow-up studies [79]. Finally, most recently, a methylation-based clock was developed and used to screen for geroprotective compounds in vitro [80]. 

Most screens for geroprotective compounds use longitudinal follow-up of death as a readout for aging. Such mortality studies have been the gold standard in aging research [81]. However, some recent research suggests that health- and lifespan can under some conditions be uncoupled [82,83,84,85]. Hence, assays that measure certain health parameters such as motility in addition to mortality have a great potential in the development of geroprotective interventions. 

A major drawback of phenotypic screening is the exclusion of potential leads with bad pharmacodynamic and pharmacokinetic properties such as poor adsorption, rapid metabolism or toxicity [47]. Leads with unfavorable pharmacodynamic and pharmacokinetic properties could still, after significant lead optimization, result in new drugs. Furthermore, lead optimization using structure–activity relationships (SAR) can be very complicated in phenotypic screening if the target remains undiscovered [47].

A recent extension of phenotypic screening is high-content screening (HCS), which uses automated imaging platforms to record multi-dimensional data such as size, shape, granularity and fluorescence of cells [86,87]. For example, a HCS drug screen was conducted in *C. elegans* to identify drugs that induce mitochondrial phenotypes that might be predictive of the longevity potential of the tested drug [74]. Four known compounds were screened that increased lifespan by inducing mild mitochondrial stress (paraquat, rotenone, doxycycline and oligomycin). It was demonstrated that, at life extending concentrations, these compounds caused a decrease in size of the worms and induced the expression of the stress reporters, validating the assay. Next, the authors used this assay to test a novel ATPase inhibitor, LYC-30904, and found that it indeed induced these mitochondrial phenotypes. The compound was subsequently tested for lifespan and found to significantly increase lifespan.

### 2.3. Target-Based Screens for Geroprotective Drugs

In the last thirty years, many genes have been identified that, when manipulated, increase lifespan in various model organisms [23,88,89]. Several of these genes are evolutionarily conserved and present in humans, and they encode proteins that are potentially druggable such as NAD+-dependent protein deacetylase sirtuin-1 (SIRT1), AMP-activated protein kinase (AMPK) and mechanistic target of rapamycin (mTOR) [90,91,92,93,94,95]. One of the earliest target-based screens for geroprotective drugs targeted SIRT1 and led to the identification of several compounds, most notably resveratrol, which activate SIRT1 [90]. Next, a high-throughput (290,000 compounds) target-based screen for SIRT-1 activators was conducted that led to the identification of various SIRT1 activators with a potency of over 1000-times that of resveratrol [91]. The level of nicotinamide adenine dinucleotide (NAD+) decreases with age [96,97,98,99,100] and it has been suggested that increasing NAD+ levels could improve health in the elderly and maybe extend lifespan [101,102,103]. Multiple pathways consume NAD+ in vivo but especially the CD38 enzyme may be responsible for the age-related decline in NAD+ levels [104,105]. At GlaxoSmithKline, a HTS campaign for CD38 inhibitors was set up, which resulted in the identification of various weakly active inhibitors. SAR optimization studies led to the identification of several potent CD38 inhibitors that were demonstrated to increase NAD+ levels when given orally to mice [106,107]. Furthermore, one of these inhibitors was shown to improve various markers of health in mice including glucose tolerance, muscle function, exercise capacity and cardiac function [108].

## 3. Important Considerations for Geroprotective Drug Screening in *C. elegans*

One of the most important parameters in drug development is the absorption, distribution, metabolism, excretion and toxicity (ADME-Tox) of leads. In target-based drug development ADME-Tox considerations are only made after the initial screen, namely during the lead selection and optimization phase [109]. However, in phenotypic drug discovery ADME-Tox, properties must be considered even before conducting the screen. Here, we briefly discuss the ADME-Tox considerations as well as some broader considerations in the development of reproducible phenotypic drug screens for geroprotective drugs specifically in *C. elegans*.

### 3.1. Pros and Cons of Drug Screening in C. elegans

The free-living nematode *C. elegans* was first introduced as a model organism by Sidney Brenner in 1963 [110]. Since then, it has become a very popular model organism due to its many advantages. Its small size (adult size ~1.5 mm), high fecundity (300 offspring per unmated hermaphrodite), fast generation cycle (~3 days from egg to adult at 25 °C) and cheap culturing conditions make it an economical model organism that is ideally suited for high throughput studies. Furthermore, its completely sequenced genome, the availability of large mutant collections and RNAi libraries that cover nearly its complete genome and the ease by which it can be genetically engineered make it a very powerful model for many forms of research [111]. These genetic tools also simplify the investigation of targets and mechanisms of drug hits [112]. The ability to store strains indefinitely in liquid nitrogen makes it very economical to maintain large mutant collections and prevents genetic drift during continuous subculturing [113]. Despite the large evolutionary distance between *C. elegans* and humans, 41% of the *C. elegans* genes have human orthologs [114]. Even more remarkable is that up to 75% of disease-related genes have *C. elegans* orthologs [115]. Indeed, several pathways modulating the aging process have proven to be highly conserved from *C. elegans* to humans [20,24]. Furthermore, aging *C. elegans* show multiple pathologies that are similar to those observed in elderly humans such as loss of muscle function (sarcopenia), loss of learning ability and increased risk for infectious disease [116,117]. The worm also amends itself to the creation of human disease models such as Alzheimer’s disease [118,119], Huntington’s disease [120], amyotrophic lateral sclerosis [121], Duchenne muscular dystrophy [122,123] and hereditary transthyretin amyloidosis [124]. These disease models have been used in multiple drug screens. The study of anatomical defects in *C. elegans* is facilitated by the invariant cell lineage [125,126]. Furthermore, its optical transparency allows for in vivo observation of morphology, pathological changes and fluorescent or luminescent reporters [127]. *C. elegans* has also proven to be an excellent model to study toxicity. In general, the ranking of compounds for toxicity in *C. elegans* closely matches that observed in mammals [128]. A major benefit of phenotypic drug screening in *C. elegans* is that toxic leads can be eliminated early on in the drug development pipeline. Furthermore, drug metabolism is remarkably similar between *C. elegans* and mammals with Phase I and II enzyme modifications to drug compounds [129].

Because of the many advantages that *C. elegans* offers as a model organism, it has become the most popular model in geroprotective drug screening. This is illustrated by the fact that almost 70% of drugs listed in the DrugAge database have been tested in *C. elegans* compared to just 10% in mice (https://genomics.senescence.info/drugs/stats.php). However, surprisingly few geroprotective drug hits first identified in *C. elegans* have been validated in other model organisms. One example of a geroprotective drug first identified in *C. elegans* [130,131] and later validated in *Drosophila* [132] is lithium. In contrast, many of the well-known geroprotective drugs such as biguanides were first identified in rodents [133,134,135] and only later shown to also extend lifespan in *C. elegans* [136,137,138,139]. The high cost of lifespan studies in mice means that only the most promising leads from screens can advance to this stage. We recommend validating the *C. elegans* hits first in cheaper organisms such as *Drosophila* or turquoise killifish before advancing to mammalian lifespan studies [140,141]. 

*C. elegans*, as with all model organisms, has several limitations as well. For example, it lacks many tissues and organs present in humans such as the heart, kidney, liver, lungs and eyes [128,142]. In addition, the intestine in *C. elegans* is the major organ for detoxification and fat storage functions that in mammals are carried out by the liver and adipose tissue, respectively [143]. It also lacks an adaptive immune system, and, while *C. elegans* does have an innate immune system, it differs in several key ways from the mammalian innate immune system such as the apparent lack of any nuclear factor-κB (NF-κB), myeloid differentiation primary response 88 (MYD88) and cytokine homologs [144]. These factors limit the use of *C. elegans* as a model to study inflammation and immunosenescence. However, *C. elegans* does experience an increase in susceptibility to bacterial infection with age [117]. *C. elegans* is fully postmitotic excluding its use for the study of stem cell aging and tissue repair. Telomere length is very variable and no correlation exists between telomere length and lifespan in *C. elegans*. Furthermore, telomeres in *C. elegans* do not shorten with age [145]. Another consequence of the lack of mitotic cells in *C. elegans* is that it is unlikely that senescent cells will accumulate with age. However, it should be pointed out that postmitotic cells can go into a “senescent cell-like” state [146] and that worms stressed by salt show an increase in β-galactosidase staining [147,148], a classical marker of cellular senescence. However, to establish that *C. elegans* cells can undergo cellular senescence would require the measurement of multiple senescence markers [149], something that, to the best of our knowledge, has not been done yet. Indeed, SA-β-galactosidase staining is not sufficient to prove cellular senescence as quiescent macrophages have also been found to be SA-β-galactosidase positive [150]. Many of the classical senescence markers [151] are also not amenable to *C. elegans*. For example, *C. elegans* has no ortholog of the p16^INK4a^ protein. In addition, the use of other cell cycle markers is not possible due to the postmitotic nature of the *C. elegans* soma [152,153].

Another limitation of *C. elegans* is its small size, making it hard to obtain adequate quantities of biomass, especially of older worms, for biochemical assays [154]. Moreover, it is difficult to isolate individual cells or tissues for biochemical or gene expression analysis. However, it is possible to cultivate isolated cells from embryos or larval stages and induce their in vitro differentiation [155,156,157]. In addition, FACS sorting of cells obtained from dissociated larvae can be performed [158,159]. The lack of commercial antibodies against most *C. elegans* antigens combined with the fact that many proteins in *C. elegans* are divergent enough from their vertebrate counterparts to preclude the use of vertebrate antibodies is also a disadvantage of this organism [160].

Finally, *C. elegans* has evolved specific adaptations to its ecological niche that are not conserved in mammals such as the dauer diapause [161,162]. The fact that *C. elegans* exists as populations of self-fertilizing hermaphrodites may have important implications for the evolution of their longevity. In fact, it has recently been suggested that in *C. elegans*, in contrast to most other species, aging may have evolved as an adaptation [163,164,165].

### 3.2. Genetic Background of C. elegans

First, the genetic background of the worm in which the screening takes place should be precisely known. For example, the Bristol N2 hermaphrodite strain from CGC is generally considered the wild-type (WT) strain, but in 2018 a user reported that this N2 strain contained the *alh-2(ot588)* mutation (https://cgc.umn.edu/strain/N2). Recently, the Gems lab reported that the Bristol N2 male stock from CGC contains the *fln-2(ot611)* mutation [166]. These findings have been confirmed by our lab (unpublished findings). To avoid genetic drift, it is recommended to regularly thaw new stock. Newly thawed worms should be cultured for at least three generations after thawing before being used in experiments to remove epigenetic marks caused by the freezing and thawing procedure [167]. 

### 3.3. Effect of Bacteria on Administered Drugs

**Figure 1:**
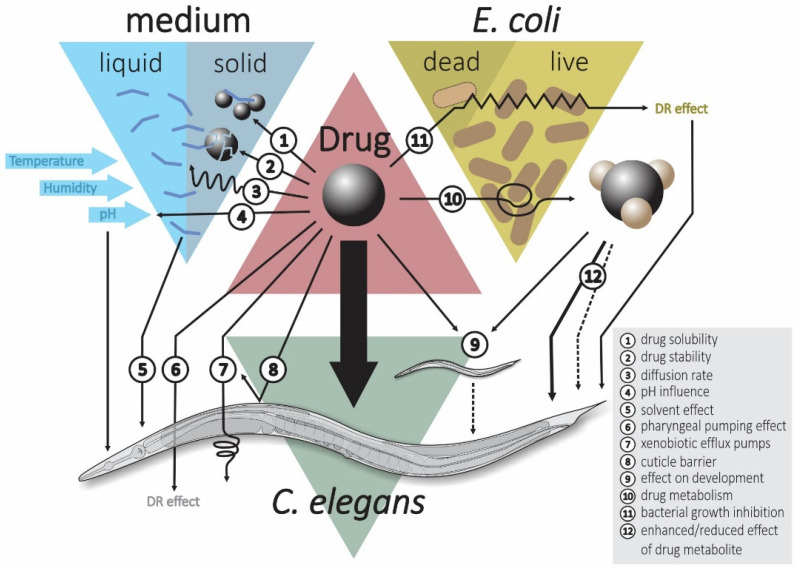
The influence of various biotic and abiotic factors on drug screening in *C. elegans*.

Most laboratory studies use live *E. coli* OP50 rather than killed bacteria as the food source for the worms [168,169]. This method introduces the complexity that bacterial metabolism of the drug compounds can decrease the drug efficacy by conversion of the drug into inactive metabolites, activate the drug leading to higher activity or side activities (e.g., toxicity) can occur (Figure 1) [170]. The interference of the bacteria on drug action can be avoided by using dead bacteria (e.g., heat killed) [171]. Indeed, it has been shown that the drug concentration in the worm’s body is higher when grown on dead bacteria compared to live bacteria [172]. Consequently, several geroprotective drug studies in *C. elegans* have used heat killed bacteria as the food source [173,174,175]. Nevertheless, the use of dead bacteria also carries downsides, most importantly, *C. elegans* growth is strongly impaired leading to a delay in development [176]. Moreover, worms grown on arrested or dead bacteria live longer [177,178,179] and this may prevent the discovery of drug hits that act through similar mechanisms. Furthermore, the human gut microbiome may be important for the action of several drugs [180,181], and, hence, it is likely more relevant to study the effect of orally administered drugs in *C. elegans* in the presence of live bacteria. Metformin was found to increase lifespan of *C. elegans* when grown in the presence of living *E. coli* OP50 while actually reducing lifespan when grown on dead *E. coli* OP50 [137]. However, the metabolic capacity of a single bacterial species in the *C. elegans* microbiome is still very different from the metabolic diversity of the human gut microbiome.

### 3.4. Drug Administration

Another important variable is the method by which the drug is administered to the worms. In solid nematode growth medium (NGM) lifespan experiments, the drug can be either mixed into the molten agar or later added as a solution on top of the solidified agar. Factors such as humidity, interactions of the drug with the agar and solubility of the compound can all potentially influence the real level of exposure of the worms to the drug. In contrast, liquid medium provides a much more controllable dosing environment [128]. Supplementing the drug into molten NGM may cause degradation of the compound due to the high temperature [182]. Another potential problem with the addition of drugs to molten NGM is that the drug will be present when the OP50 lawn is grown on the plate. In addition to the risk that the drug gets metabolized before worms are added (see above), there is also the risk that the bacterial growth is affected by the presence of the drug leading to an unwanted dietary restriction effect. Indeed, studies have found that bacterial growth is impaired on NGM plates that contain 5-fluoro-2-deoxyuridine [183,184] or metformin [137]. One study investigated five different methods of drug administration to worms and found that the highest absorption was achieved when the drug was mixed with the molten NGM and the resulting plates were seeded with dead *E. coli* OP50. In addition, supplying the drug in liquid medium containing dead bacteria resulted in high drug concentrations in the worm. Adding the drug as a solution on top of solidified NGM plates followed by the addition of dead bacteria resulted in a medium level of drug uptake. Finally, mixing the drug in the molten NGM and seeding the plates with live bacteria or mixing the drug with live bacteria and spreading that solution on plates resulted in the lowest drug concentrations in the worms [172]. If the drug is added as a solution on the surface of the agar plate, it will inevitably take time for the drug to diffuse through the solid medium. Not much research has been done on this but some researchers incubate plates, on which the drug has been added as a liquid solution, for 6–24 h before transferring worms [185,186]. Drug uptake by worms grown on NGM plates is sometimes absent while that same drug is taken up when supplied in liquid medium. This observation can be explained by noting that NGM is a highly complex mixture of two undefined compounds (agar and bactopeptone) that could interfere with drug uptake. The high concentrations of small molecules present in bactopeptone could decrease the solubility of drugs in NGM, leading to precipitation of the drug in the plates, which can sometimes be visually seen as “crystal gardens” [187].

Stock solutions of compounds used for HTS are typically prepared in DMSO as this generally solubilizes the majority of drug-like compounds. Cell lines typically do not experience negative effects from 0.1% DMSO while biochemical assays can tolerate up to 1–5% DMSO [51]. In *C. elegans,* DMSO has been found to increase mean lifespan in a dose-dependent manner from as little as 0.01%, reaching a maximum effect at 0.5% [188]. Similarly, it was found that 0.8–1% DMSO increases lifespan of *C. elegans* in liquid medium by 15% [189]. In contrast, high DMSO concentrations can increase mortality [190]. In this study, various drugs were tested at different concentrations, and it was observed that for some drugs mortality was increased at the highest tested concentrations. Further study showed that it was not the drugs but rather the high DMSO concentrations that was driving the increased mortality. Therefore, control worms should receive the same solvent exposure as the drug-treated worms [191]. Interestingly, resveratrol failed to extend lifespan in the absence of DMSO, while lifespan was extended in the presence of DMSO, probably because DMSO is needed for solubility [192].

### 3.5. Drug Stability

Many organic molecules experience significant degradation upon prolonged storage as solutions [193]. DMSO is hygroscopic and thus readily absorbs water from the air leading to a decrease in compound concentration over time, a change in compound solubility, an increased rate of compound breakdown and a decrease in melting point [193,194]. The melting point of dry DMSO is 18.5 °C and hence solutions can be frozen by storing them at 4 °C (or at −20 °C to further reduce compound breakdown) [194]. The stability of 778 compounds in 100% DMSO for six months was investigated under various storage conditions and then the stability was extrapolated for the next four years leading to a degradation of 12%, 21% and 58% for compounds stored at −20 °C under argon, −20 °C under air and at +15 °C under argon, respectively [195]. For example, it was noted that darkening of a thioflavin T stock solution was associated with a loss of the lifespan extending effect and even caused early deaths [196]. Presumably, the darkening was a result of the gradual degradation of the compound over time. In addition, repeated freeze/thaw cycles could affect the stability of compound solutions. Interestingly, no additional peaks were observed in the HPLC chromatograms leading the authors to speculate that the loss of compound may principally be driven by precipitation [193]. Indeed, a precipitate was observed in many of the solutions at the end of the study. Finally, it should be noted that bioactive molecules that can interfere with assays can leach from the plastic storage containers and that this problem likely worsens with increased contact time between the plastic and the solution and with increased temperature [197,198,199]. 

### 3.6. Age of First Drug Exposure

The age at which drug treatment is started may also be an important variable to consider. The administration of drugs during larval stages may interfere with normal development. Furthermore, if a drug is administered during the early larval stages, it could potentially induce dauer formation [128]. Valproic acid extends lifespan when administered from conception [200], but, in another study, where it was administered during the adult phase, no effect on lifespan was found [186]. When the effect of 21 lifespan extending compounds was tested starting from either Day 1 of adulthood or Day 8 of adulthood, it was found that all compounds extended lifespan when started at Day 1 but only one extended lifespan when administered from Day 8. Two compounds even caused a significant decline in lifespan when administered from Day 8 onwards [201]. In a recent study, metformin was administered starting at different time points during the worm’s lifespan. At Days 1 and 4 of adulthood, metformin, at all tested concentrations, extended the lifespan of the worm while at Day 10 it reduced lifespan at all tested concentrations [202]. At Day 8, the effect on lifespan was dose dependent. Similarly, when metformin was administered to SHR mice from the age of three and nine months, it extended mean lifespan by 14.1% and 6.1%, respectively, but no effect was seen in mice administered metformin from age of 15 months. Furthermore, the mean lifespan of tumor-free mice started on metformin from the age of 15 months was significantly decreased by 12.8% [203].

### 3.7. Drug Uptake

*C. elegans* has an impermeable cuticle that forms a strong barrier for the absorption of many drugs. Mutants have been created that have a more permeable cuticle, which should allow for easier absorption of exogenously added compounds in drug screening and toxicity testing [204,205,206]. *C. elegans* also has an extensive enzymatic detoxification system and likely contains xenobiotic efflux pumps [207]. All this makes that drug absorption in *C. elegans* is notoriously limited. For example, it was discovered that fewer than 10% of the over 1000 compounds tested were able to accumulate in the worm to concentrations over half of that present in the medium. The authors used this knowledge to build a predictive model for drug uptake in *C. elegans* based on the chemical substructures present in the drug molecule [129]. To overcome uptake problems, studies in *C. elegans* often use very high drug concentrations compared to studies in mammalian cell culture. However, the use of high drug concentrations adds expense to the screen and could lead to problems such as a lack of solubility of some compounds [129]. In addition, if only one concentration is tested, then the use of high concentrations may lead to high levels of false negatives due to off-target toxicity. A possible solution to this problem is to retest all compounds that show toxicity at a lower concentration [63]. To increase uptake efficiency of hydrophilic compounds, they can be loaded into liposomes before administration to *C. elegans* [208]. 

### 3.8. Food Intake

An important confounder in geroprotective drug screening is the influence of a drug on food intake [209]. Reduced food intake has been found to increase lifespan in many model organisms including *C. elegans*, mice and rats [17,18,19,210,211]. Thus, any drug that reduces the rate of pharyngeal pumping will likely extend lifespan simply by reducing the ability of the worm to eat. Hence, food intake assays [212] should be performed on all positive hits from these drug screens. Another important consideration for conducting lifespan tests is that the method must be optimized to ensure that, at no point in time during the lifespan assay, food concentrations become too low and induce a dietary restriction effect. Especially, when culturing worms in small volumes such as 96- or 384-well plates, food levels can become critically low, if the assay protocol has not been properly optimized before starting the screen.

### 3.9. Abiotic Factors

*C. elegans* is exquisitely sensitive to a wide range of environmental factors such as temperature [213], humidity [214], light exposure [215] and population density [216]. In fact, it has been demonstrated that the growth rate of *C. elegans* larvae was significantly accelerated by simply wrapping the Petri plates, in which they are cultured, with Parafilm M^®^ [217]. It is also worth noting that the effect of interventions can be temperature dependent. For example, *rsks-1(ok1255)* mutants have an increased lifespan compared to wild type at high temperature, but actually live shorter at low temperature [218]. The common range of temperatures for growing *C. elegans* in the lab ranges from 15 to 25 °C, whereas most lifespan experiments have been conducted at 20 °C [219]. 

Some drug compounds may also strongly change the pH of the medium, thereby affecting the effect of lifespan. For example, in a study on the effect of α-ketoglutarate on lifespan in *C. elegans,* it was observed that the stock solution of α-ketoglutarate had a pH of 1.56, which might overwhelm the buffering capacity of the NGM, leading to an abnormally low pH of the medium. However, adjustment of the pH by titration with NaOH did not influence the lifespan results obtained by α-ketoglutarate treatment [220].

### 3.10. Reproducibility and Plate-to-Plate Variability

Before a full screen is conducted, a smaller pilot screen should be conducted to verify the quality of the assay. A variety of positive and negative controls should be included in this pilot screen. Positive controls for *C. elegans* lifespan studies include compounds with known longevity extending effects such as rapamycin or RNAi or mutants of well-known longevity genes such as *daf-2* or *eat-2*. As a negative control, knockdown or mutants in *daf-16* could be used. Typically, in HTS, the assay quality is judged by the Z’ factor. A Z’ factor value between 0.5 and 1 is considered excellent, while a value below 0.5 indicates that the assay is of moderate to poor quality and may need optimization before it can be used in HTS. In case of a sub-zero Z’ factor the assay cannot be used in HTS [221]. Furthermore, the Z’ factor is often used during a screening campaign to monitor assay performance [222].

In screens employing multiwell plates, it is important to consider systematic errors resulting from well location due to the inherent heterogeneous spatial design of the plate. In particular, wells at the periphery of the plate are susceptible to systematic bias, so-called “edge effects”, due to for example evaporation and temperature inhomogeneities [223,224,225]. Other still uncharacterized factors may also play a role such as differences in gas exchange [224]. Therefore, some decide to avoid using edge wells at all during screenings [226]. In addition, the addition of distilled water in the void between the wells can reduce evaporation and thermal gradients [227]. Controls should ideally be located randomly within a plate but most often they are placed in the first and last column of the plate. In that case, the influence of the edge effect on these controls can be minimized by alternating positive and negative controls [228,229].

After a screen is successfully conducted, the positive hits should be validated and quality control experiments should be conducted. For example, after an automated lifespan screen (see below), the hits could be validated by manual lifespan assays and dose–response experiments can be conducted. The chemical identity and purity of the compounds used should also be checked [46].

Finally, it is important to notice that reproducible results can only be obtained when all methods used are thoroughly standardized between experimental runs. Very minute differences such as the way worms are picked from a plate and transferred to a new one can greatly influence the outcome of an experiment [230]. The Caenorhabditis Interventions Testing Program has published detailed protocols for lifespan experiments in *C. elegans* [167]. Using these standardized protocols, it was demonstrated that variation between three different labs in the effects of ten compounds on lifespan was remarkably minor. In fact, using a general linear model, it was found that just 1% of variation in the results was attributed to between lab variation [186]. Several other excellent reviews discuss potential sources of variation and offer suggestions to improve reproducibility of research in *C. elegans* [171,231].

## 4. Limitations of Manual Lifespan Assays

Manual lifespan assays when working with worms can either be performed on agar plates or in liquid medium.

### 4.1. Agar Plate-Based Lifespan Assays

The standard way of conducting an agar plate-based lifespan assay in *C. elegans* is by manually prodding the worms on a regular basis (either daily or every other day) with a platinum wire or eyelash and classifying animals as alive or dead based on movement. In addition, animals that die from non-aging related causes are generally censored, meaning they are excluded from the analysis. This includes animals that are missing, are accidentally killed by the researcher, desiccated worms on the walls of the dish, worms burrowed in the agar and the occurrence of internal hatching or vulval rupture [219]. Conducting manual lifespan assays in *C. elegans* is a repetitive, labor intensive process that is subject to observational bias. Observer fatigue could induce variability between plates [232]. Furthermore, the repeated manual prodding could result in mechanical damage, especially in old fragile individuals, and consequently shorten survival. Indeed, Pitt et al. (2019) observed an even longer lifespan in long-lived *daf-2(RNAi)* worms assayed by an automated lifespan system compared to a manual assay [233].

Another important consideration with manual assays is that, during the first 7–10 days of adulthood, animals have to be moved to new plates on a daily basis to prevent offspring contamination. The use of temperature-sensitive sterile mutants, such as *glp-1*(e2141), *glp-4(bn2)* or *fer-15(b26); fem-1(hc17)*, or the addition of 5-fluoro-2-deoxyuridine (FUdR), which inhibits reproduction, significantly reduces the labor burden of the lifespan assay. Temperature-sensitive sterile mutants are fertile at a permissive temperature (15 or 20 °C) but sterility is induced upon cultivation at a higher restrictive temperature (25 °C) [219]. Notably, *glp-1* has a longer lifespan compared to wild type N2 worms, and this increased longevity is dependent on DAF-16 [234,235]. Since various geroprotective drugs depend, in part, on DAF-16 for their effect on lifespan [236,237,238], these drugs would have been missed in *glp-1* screens as they would not be able to induce a greater level of DAF-16 nuclear translocation than what is already achieved by the *glp-1* mutation. As a consequence, few studies have used temperature-sensitive sterile mutants for geroprotective drug screening [131]. FUdR prevents the hatching of eggs and is typically used at concentrations between 25 and 120 µM [239]. However, while FUdR had no effect on lifespan of wild type worms at 20 °C, it has been found to increase mean lifespan in wild type worms grown at 25 °C [240] and in certain mutants [241,242]. Additionally, it has been reported that FUdR activates stress response pathways [240,243] and increases fat accumulation [241]. It has been reported that in some plant extract screenings, larvae were observed when FUdR was used at low concentrations (≤ 50 µM) possibly due to interference of the plant extract with the molecular effects of FUdR [171]. Therefore, these authors started to use FUdR concentrations of 200 µM and even up to 500 µM in compound screens. However, FUdR is expensive so the use of high FUdR concentrations in a screen would be a significant cost factor.

### 4.2. Liquid Culture-Based Lifespan Assays

*C. elegans* can be grown in liquid culture in the absence (axenic) or presence (monoxenic) of *E. coli* as a food source. In this article, we only discuss the use of monoxenic liquid culture as, to the best of our knowledge, no large-scale drug screens in axenic culture have been performed. The lab of Michael Petrascheck has screened 88,000 compounds in liquid monoxenic culture in 96- and 384-well plates, leading to the identification of 115 compounds that significantly increase lifespan [244]. This technique offers several benefits over classic solid medium based assays. A major benefit of liquid culture screening is that many automated liquid handling robots for multiwell plates are commercially available. For smaller academic labs that do not have this equipment, the use of multichannel pipettors can also greatly reduce labor compared to solid agar-based lifespan assays. Furthermore, liquid screens use less drug compound, which can be a significant cost factor [187]. In addition, compounds may not be available in larger quantities. For example, commercial compound libraries are typically supplied at low milligram quantities.

However, liquid culture also has several drawbacks. First, lifespan of worms grown in monoxenic liquid culture may be different from those grown on solid medium [179]. Worms grown in liquid culture are longer [245] and have a higher incidence of internal hatching [246] compared to NGM agar. Liquid culture also induces changes in gene expression [247,248]. Moreover, the health of the worm may be adversely affected by continuous swimming in liquid medium. While the effect of continuous swimming has not been studied, transient exercise has been shown to induce oxidative stress and fatigue during the exercise period [248,249]. While such short-term exercise regimes were shown to lead to health benefits probably through hormetic action [248,249,250,251], continuous exposure to the stress of exercise would be expected to have detrimental consequences. In addition, bacterial oxygen consumption can result in oxygen depletion in liquid medium leading in the most extreme case to the death of the worms [252]. Worm transfers are very complicated in liquid culture, hence the use of FUdR or sterile mutants is required when using liquid medium [239], except in cases involving certain microfluidics devices (see below). Finally, it should be noted that discrepancies in the lifespan effects of drugs on worms grown in liquid culture versus on NGM plates have been observed. As one example, the drug Mianserin extended lifespan in liquid culture [244] but shortened lifespan on NGM plates [253].

### 4.3. Methods to Increase Throughput of Lifespan Assays

Various modifications to the way that manual lifespan assays are being conducted have been implemented to increase throughput. One method is the replica set method (RSM), in which representative replicates are kept for each time point at which survival will be scored, in contrast to the longitudinal follow-up of the same population that is used in traditional lifespan assays. The throughput is increased because the replicate plates can be flooded with buffer causing living worms to swim, making differentiation of dead and living worms much faster than touching them with a worm pick. After counting, the plate is discarded [254]. Alternatively, workload can be reduced by regularly scoring the negative control wells until >95% mortality in those wells is observed. When this time point is reached, the drug-treated wells are examined and only wells in which live worms remain are scored. Using this method, one study group was able to screen 30,000 compounds, an effort that resulted in the identification of 500 primary hits, 180 of which were selected for re-testing and ultimately led to the identification of 57 compounds that reproducibly increased longevity. The authors of this work also noted that it may be useful to re-score the drug wells at the time point when >99% mortality is reached in the control wells. Furthermore, if the screen is especially high throughput, labor can be reduced by performing the initial scoring at the >99% mortality time point [255,256].

A common strategy used in both target-based and phenotypic drug screening to increase throughput is to pool compounds. For example, in a screen for compounds that enhance neurogenesis, 1000 compounds were tested by injecting pools of ten compounds in the ventricles of the brain of living mice. Each pool was injected in two mice, hence the whole screen used only 200 mice [257]. However, such a multiplexing strategy carries various risks such as chemical interaction of the pooled compounds, solubility problems, and possible synergetic or antagonistic effects between pooled compounds [258,259,260]. For example, the presence of an acutely toxic compound in the pool would be expected to completely mask the beneficial effect of a geroprotective drug present in the same pool. One solution that has been employed to overcome these limitations is to include each compound in more than one pool [261].

Manual assays are also sensitive to variations in environmental conditions. For instance, the plates need to be removed from the incubator for scoring, potentially resulting in unintended exposure to environmental contaminants. In addition, as the lifespan of *C. elegans* is very temperature sensitive, variations in external temperature can influence the experiment [213]. In contrast, automated lifespan devices can be kept for the whole duration of the experiment in a temperature-controlled incubator, eliminating the influence of temperature or environmental variation between conditions and replicates [262]. In a study on the automated NGM based WormBot platform, no well to well variation in lifespan was observed and it was demonstrated that the average temperature variation between the wells and the surrounding environment remained limited to just 0.04 °C [233]. *C. elegans* lifespan is also sensitive to light exposure [215], and light exposure can be quite variable during manual scoring if not properly controlled by the investigator. While the overall level of light exposure is higher in typical automated lifespan set-ups, the exposure is at least very consistent between conditions and replicates.

### 4.4. However, Manual Assays also Have Some Strengths

While manual lifespan assays suffer from various limitations (as detailed above), it should be noted that they also offer several strengths compared to automated lifespan devices (discussed below). For example, because the plates are monitored manually, deviations could be spotted such as contamination, food depletion or drying out of the plate. It also allows for censoring of worms that die from non-aging related causes. In automated systems, such censoring is only possible in case of manual post-hoc data analysis. Examination of the plates by eye could also uncover unexpected phenotypic differences (such as differences in movement) or differences in health. Manual lifespan assays allow for complex designs such as transfer of the worms to new drug treated plates at specified moments or switches between liquid and solid medium. However, probably the most significant benefit of manual lifespan assays is that the use of FUdR or sterile mutants to avoid generational mix-up can be completely avoided by applying manual transfer of worms to fresh plates.

## 5. Automated Phenotyping and Lifespan Devices 

Because of the limitations of manual survival assays, automated methods for lifespan determination have been developed. These automated lifespan assays offer several distinct benefits over manual assays, such as increased temporal resolution and an increased number of technical and biological replicates that can be run (Table 2). However, they may also introduce new challenges such as temperature gradients produced by various electronic and electromechanical parts. This may especially become a problem if the goal is to miniaturize the assay as much as possible (for example, growing worms in 384-well plates).

Contamination can be a risk in long-term cultures and hence many studies that use automated lifespan devices add antibiotics and/or antimycotics to plates, wells or microfluidic chips to prevent bacterial or fungal contamination [232,233,263,264]. The use of the streptomycin-resistant *E. coli* OP50-1 strain allows the addition of streptomycin to the medium to prevent bacterial contamination [265]. However, one should be wary of adding extra chemicals such as antibiotics or antimycotics to *C. elegans* cultures, as these could interfere with the experiment in unexpected ways. Rigorous maintenance of sterile technique is the most important recommendation to prevent contamination.

One of the biggest hurdles to the development of automated HCS platforms for *C. elegans* is automated image capturing and processing. Software must be able to distinguish worms from debris and, even more challenging, to “untangle” clusters of worms. Worms expressing a fluorescent protein in their pharynges can be used to enhance discrimination of individual worms [232].

In manual lifespan assays, death is determined by failure to respond to touch-provoked movement. However, this metric is not possible in automatic systems. Such systems generally depend on the determination of the last time point at which a worm still showed spontaneous movement [262] or by inducing movement through a short flash of blue light [263].

New automated systems should always be validated by comparing lifespans with those obtained with manual assays. If any deviations between the two methods are found, then in depth studies should quantify which factors are responsible for the observed differences. Furthermore, trial-to-trial variability should be measured to investigate reproducibility of the method [266].

We broadly divide the various automated lifespan devices in two main categories: (1) those based on microfluidic chips; and (2) those based on solid agar culture. We discuss stress-based screens in a separate heading but most of those methods are based on either microfluidic or solid agar lifespan devices except for one notable exception, the recently developed LFASS assay.

### 5.1. Microfluidics-Based Platforms

Microfluidic worm chips are made from polydimethylsiloxane (PDMS). PDMS is a type of “silicone” that is non-toxic, chemically inert, permeable to oxygen and carbon dioxide and optically transparent for wavelengths over 230 nm, allowing for brightfield and fluorescence microscopic imaging of the worms. PDMS adheres tightly to glass surfaces, and hence worm chips are commonly mounted on glass microscope slides. The chips are produced by a soft lithography technique. Food can be added at user defined time points (e.g., once a day) [246,267] or continuously through the use of automated pumps [264].

Various microfluidics chips for lifespan assays have been developed including Lifespan-on-a-chip [246] and WormFarm [264]. The Lifespan-on-a-chip device consists of an array of chambers that house individual worms. This device allows for the longitudinal monitoring of individual worms [246]. Most recently, a microfluidics device called NemaLife was designed that includes a micropillar arena. This arena allows the worms to crawl as they do on agar plates, rather than swim as in typical microfluidics devices [267]. This device also contains sieve channels, but, because the adults generally remain in the micropillar area, the risk of physically damaging them is reduced. Furthermore, this device allows for the evaluation of two healthspan markers, pharyngeal pumping and vibration-induced locomotion, assays that have to be conducted manually [267]. A commercialized version of the NemaLife microfluidics chip combined with a benchtop machine that regulates fluid flow through the chip and enables time-laps image recording and analysis has been developed (https://www.nemalifeinc.com/). Similarly, a device called the stress-chip combines crawling and swimming areas [268]. It should be mentioned that, to the best of our knowledge, no large drug screens in microfluidic devices have been conducted so far. We refer the reader to two excellent reviews on the various microfluidic platforms for HTS in *C. elegans* [266,269].

One downside of these microfluidic devices is that bacteria can get stuck to the walls, obscuring the visualization of the worms and resulting in clogging of the channels. Coating the inside of the chip with 2-[methoxy(polyethyleneoxy)_6-9_propyl] trimethoxysilane, or Pluronic^®^ F-127 reduces the adhesion of bacteria to the channel walls [246,270,271]. Clogging can also occur due to precipitation of bacteria in the inlet reservoir. This can be avoided by replacing the bacterial suspension in the inlet reservoir daily. The fresh bacteria suspension should be filtered, to further remove any particulates that could clog the microfluidic channels, prior to loading in the inlet reservoir [246,271]. Some investigators have also placed a tiny magnetic stir bar in the syringe containing the bacterial suspension to prevent precipitation of the bacteria [270]. As noted above, contamination is a potential risk in long-term *C. elegans* culture in microfluidic devices. These PDMS microfluidic chips can be sterilized by autoclaving [269], a 5-min rinse with 70% ethanol [267] or by a combination of ethanol and UV treatment [272]. In addition, as explained above, antibiotics and/or antimycotics are frequently added to reduce contamination risk [265]. Microfluidics devices can also be used in combination with sterile axenic medium, which prevents bacterial metabolism of drugs (see Section 3.2) as well as biofilm formation inside the microfluidics device [272]. A major limitation of microfluidics technology is that it requires specialized equipment and expertise that is not available to most *C. elegans* laboratories. Furthermore, most aging studies have been conducted on agar plates and hence data obtained through microfluidic platforms cannot easily be compared to existing knowledge.

A major benefit of microfluidic devices is that the use of FUdR or sterile mutants can be avoided. Eggs and hatched L1s are simply washed away continuously through sieves that prevent adults from escaping. However, adult worms can get injured by being pushed against the sieve channels and these narrow sieve channels are also at risk of getting clogged [264,273]. As discussed above, the recently developed NemaLife system reduces the chance of physical damage to the worms from contact with the sieve channels [267].

Microfluidic devices offer the ability to track individual worms longitudinally, permitting insights into intra-individual variability in responses [270]. A very significant contributor to the overall cost of various systems is the quantity of chemicals and/or drugs required. Microfluidic methods greatly reduce the quantity of chemicals and/or drugs required thereby reducing the overall cost of the screen [264]. Furthermore, it was found that pharyngeal pumping rates are better maintained with age in the NemaLife microfluidics device compared to worms grown on agar, a feature that may result in better uptake of drugs by old worms [267]. In agar-based lifespan assays, a significant proportion of the population can be lost due to burrowing into the agar or crawling up the side wall followed by death due to desiccation [186]. As such deaths are censored, it becomes a question whether the lifespan data obtained from this selected subset are truly representative for the population as a whole. Microfluidics devices eliminate these two sources of death and therefore might more accurately capture whole population lifespans [267].

Liquid culture of *C. elegans* offers several distinct benefits over solid plate culture. First, the concentration of the bacterial food can be very precisely controlled in liquid culture. Bacterial concentration greatly influences the lifespan of *C. elegans* [179] and consequently drug lifespan experiments. Bacterial food concentrations and environmental conditions, such as the presence of metabolites and waste products from the worms and bacteria, change over time in agar plate-based assays. Periodic transfer of the worms to new plates can minimize changes in the environment but the transfer also introduces other problems such as loss of worms or physical damage [268]. In contrast, microfluidic systems that use continuous flow of fresh bacterial solution through the worm chambers keep food concentrations and environmental conditions constant [246]. Furthermore, they allow for downstream sampling and analysis of metabolites and chemicals (such as pheromones) as well as progeny produced by the worm [267,271].

### 5.2. Solid Medium-Based Platforms

In solid medium-based platforms, worms are cultured on NGM and tracked by flatbed scanner-based or camera-based imaging. Several flatbed-based automated lifespan machines have been developed including the lifespan machine [262], WormScan [274] and most recently an automated version of WormScan [275]. The lifespan machine is based on a modified flatbed scanner (http://lifespanmachine.crg.eu/lsm/). Flatbed scanners are cheap compared to dissecting microscopes, and their box-shaped dimensions allow for easy storage in temperature-controlled incubators [262]. A major limitation of the lifespan machine is that a modified version of NGM, in which CaCl_2_ is omitted, should be used to increase optical transparency [262]. The use of calcium-depleted NGM makes it difficult to compare the results obtained by this device with previous literature data that used regular NGM. The height of the NGM in the Petri plates also has to be controlled carefully so that the surface falls in the focal plane of the scanner [262]. The lifespan machine is not able to capture young, fast-moving worms, due to the difficulty in image capturing. However, as the worms age, their movement slows down and then the scanner is able to pick them up. The inability to detect young worms has apparently no ill effect on the reported lifespan of the population [262]. The lifespan machine exposes worms to intense blue/UV light, which, especially in the presence of photosensitizers, could influence *C. elegans* lifespan. Many drugs contain UV-absorbing moieties, such as benzene and heterocyclic rings, and are thus potentially susceptible to the introduction of phototoxicity. Drug compounds could also undergo photodecomposition that might result in the formation of toxic molecules [276]. For instance, thioflavin T extends lifespan of *C. elegans* in manual assays, but in the lifespan machine thioflavin T shortened lifespan significantly. The adverse effect of thioflavin T on lifespan in liquid cultures was reversed when a blue/UV light filter was installed on the lifespan machine [220]. Another problem is the heat produced by the scanner. The scanner used in the lifespan machine uses fluorescent bulbs, which produce more heat than LEDs. However, switching to scanners that use LEDs can reduce this problem [268]. The software for the lifespan machine is freely available and recently an improved version was released in beta (https://github.com/nstroustrup/lifespan).

WormBot is an open-source robotics platform made from consumer hardware that employs a camera attached to an XY plotter to track worms in NGM-filled twelve-well plates [233] (http://wormbot.org/). The recorded images can be analyzed by an automated software program.

WorMotel uses NGM-filled microfabricated well plates and is able to individually track 240 worms for over two months. The device is able to image the worms either continuously or intermittently, and both spontaneous and blue light-induced movement can be measured. The time of death is recorded as the last moment of movement [263]. WorMotel can also be used to evaluate activity-based healthspan measures [263,277]. Software for the WorMotel is freely available (https://github.com/cfangyen/wormotel).

Automated survival platforms require that worms are cultivated for prolonged periods of time on the same NGM plates or wells. This can lead to several problems such as dehydration of the agar, fungal growth and fogging of the plate lids. In the lifespan machine, the latter problem is circumvented by placing the plates upside down, without a lid on the glass surface of the scanner. The temperature gradient generated by the heat produced by the scanner greatly reduces fogging issues of the glass scanner surface, but, as an additional precaution, the glass scanner surface can be coated with an anti-fog coating such as Rain-X [262]. Similarly, fogging of plate lids can be a problem for systems that rely on closed plates, such as Wormbot, and one trick to alleviate this problem is the treatment of the lids with detergent solution, such as Tween-20 [278]. To prevent fungal growth nystatin is often added to the NGM [262].

The *C. elegans* Interventions Testing Program (CITP) uses Lifespan Machines for increased throughput in compound screening (https://citp.squarespace.com/). Thus far, this program, which was initiated in 2013, has evaluated more than 20 compounds and found several compounds that increased lifespan in at least one of the tested strains [186,220,230,279,280,281].

Several Contract Research Organizations (CROs), such as Magnitude Biosciences Ltd. (https://magnitudebiosciences.com/) and InVivo Biosystems (https://invivobiosystems.com/), have started to offer solid medium-based automated healthspan and lifespan assays.

### 5.3. Stress-Based Platforms

Increased stress resistance is a commonly observed phenotype of many long-lived *C. elegans* mutants [282,283,284]. This observation has led to the suggestion that stress resistance screens could be used to identify novel long lived mutants [285,286] as well as new geroprotective drug candidates [287]. Indeed, several drugs that increase lifespan in *C. elegans* have been shown to increase resistance to various stressors [288,289]. In a recent study, around 100,000 small molecules were screened for their ability to induce increased resistance to oxidative stress in a primary human fibroblast cell line. This screen resulted in a core set of 32 molecules that were subsequently tested for lifespan in *C. elegans* and resulted in the identification of nine molecules that increased lifespan [79].

Screening for increased stress resistance in *C. elegans* as a predictor for lifespan offers the benefit of substantially reducing the assay time. For example, a typical *C. elegans* lifespan experiment takes 3–4 weeks to complete, while acute stress survival screens can last from a couple of hours to several days depending on the type and severity of the stress applied. We refer the reader to a recent review covering the different stressors that can be employed in stress assays in *C. elegans* [219]. The first high-throughput stress survival assay in *C. elegans* employed the vital stain SYTOX green [290]. This membrane impermeable dye is excluded from viable cells but readily stains dead cells by intercalation in the DNA [291]. A modified version of the SYTOX survival assay was used to identify compounds with antimicrobial properties. Worms were infected with the pathogen *E. faecalis,* and a screen with over 37,000 compounds was conducted leading to the identification of 28 compounds with anti-microbial properties [292]. Importantly, the concentration at which some of these compounds improved survival in the worm was significantly lower than the concentration needed to inhibit bacterial proliferation in vitro. This demonstrates the power of phenotypic screens in *C. elegans* to identify compounds that would have been missed by more traditional screens that use growth inhibition or death of isolated bacteria as the read out. It should be noted that a small fraction of worms that appear dead by visual inspection may not be SYTOX stained, while some alive worms may be stained. This could be caused by SYTOX staining of necrotic cells in living animals, variability in the staining, autofluorescence or some compounds may increase the permeability of the membranes to the SYTOX dye [292]. Stress screens can also be conducted using automated platforms, as discussed above. For example, the lifespan machine was used to evaluate heat and *tert*-butyl hydroperoxide (t-BuOOH) stress resistance [262] and WormBot was used to investigate HCN stress resistance between different mutant strains [233].

Recently, an automated label-free, HTS platform to assay stress resistance in *C. elegans* was developed [68]. This assay is based on the naturally occurring burst of blue autofluorescence that accompanies death in *C. elegans* [293,294]. This autofluorescence can be quantified with a plate reader. This method offers extraordinarily high temporal resolution (up to one measurement every 2 min) combined with a high throughput, as 96- or 348-well plates can be used. The authors demonstrated that this setup can be employed for multiple stress resistance assays including heat, oxidative stress and bacterial pathogen stress. Hence, this approach should lend itself to not only screening for drugs that increase stress resistance, but also for anti-infective drugs and anthelmintic drugs. However, thus far, no published reports exist that have employed this assay for drug screening.

It should also be noted that microfluidics devices have been used for stress survival tests in *C. elegans* [268,273]. For example, a microfluidics device was used to test the effect of polydatin and (+/−)-*α*-Tocopherol on lifespan, both in unstressed and Cu^2+^-stressed conditions, validating this device for its use in drug screenings [273].

## 6. Future Outlook

The rate of discovery of new geroprotective drugs has increased significantly since the 1990s. This is probably the result of multiple factors including the increased use of short-lived models (such as *C. elegans*) and the introduction of screening methods with increased throughput [295,296]. An analysis of the DrugAge database suggests that many known pathways involved in aging have not yet been targeted pharmacologically, suggesting opportunities for the discovery of novel geroprotective drugs [296]. Throughput of phenotypic screens using whole organisms, such as *C. elegans*, is obviously lower compared to cell-based or target-based screens. However, in our opinion, phenotypic assays are better suited for the identification of geroprotective drugs due to the lack of validated targets. While hundreds of genes that influence the aging process have been found in small model organisms, only a few genes have, thus far, been validated in humans [297,298].

However, the use of automated phenotypic screening of small model organisms comes with some challenges. A major challenge in automated image-based *C. elegans* survival assays is the difficulty for the software to distinguish individual worms in worm clusters [232,299]. Furthermore, when the image data from WormBot were analyzed using a fully automated software program, it resulted in longer survival compared to manual annotation of the images or traditional hand scoring [233]. The reason for this discrepancy is not yet known. In addition, the data storage capacity required for HCS in *C. elegans* should not be underestimated [233].

Another major challenge in the field remains the validation of the numerous lifespan-extending drugs that have been discovered in *C. elegans* in other models such as rodents. In addition, as life- and healthspan can, under certain conditions, be uncoupled, it will be paramount to investigate the effects of newly identified geroprotective drugs on healthspan [82,83,84,85]. Such investigations are severely lacking as lifespan studies vastly outnumber healthspan studies [300]. Various healthspan measures have been developed in *C. elegans*; most of them are based on locomotion such as maximum speed, number of body bends and bending angle. [82,84,301]. Therefore, the development of systems that incorporate the automatic quantification of both healthspan parameters and lifespan will be paramount. Some microfluidics-based [302] and NGM-based [233,277] automated screening platforms already enable the simultaneous determination of lifespan and healthspan.

The development of geroprotective drugs by aid of using model-based screens has the potential to deliver significant economic and societal benefits [303,304].

## Figures and Tables

**Table 1:** The strengths and weaknesses of target-based versus phenotypic screening in drug discovery. SAR, structure–activity relationship.

	Target-Based Screening		Phenotypic Screening in Cells		Phenotypic Screening in Small Organisms (e.g., *C. elegans*)	
	Strengths	Weaknesses	Strengths	Weaknesses	Strengths	Weaknesses
**Target**	Known target selected for screen.	Cannot find new targets.	Target agnostic.	Target identification can be cumbersome.	Target agnostic.	Target identification can be cumbersome.
**Human relevance**		In vitro study on isolated targets.	In vitro but on whole cells. Cells used can be of human origin. Even patient-derived primary cells or in vitro reprogrammed cells from patient-derived fibroblasts.	Access to disease relevant cell types can be difficult. Diseases cannot always be easily recapitulated in isolated cells because they depend on interactions of various cells and/or systemic factors.	In vivo, small organisms contain multiple cell types and even organ systems thus better capturing disease processes that depend on cell interactions and/or systemic factors.	Small model organisms may not fully capture human biology.
**False positives**		False positives due to nonspecific mechanisms (fluorescence quenching, aggregation).		False positives due to compounds that target generic mechanisms such as protein synthesis which affect the assayed phenotype but are not specific enough to be used as drug leads.		False positives due to compounds that target generic mechanisms such as protein synthesis which affect the assayed phenotype but are not specific enough to be used as drug leads.
**Hit identification**	Will identify all hits that modify the target of interest.	Hits will include molecules that cannot be used as drug leads (such as cytotoxic compounds).	Initial screen may already inform about toxicity of compounds (cell viability). Hits already have “drug-like” properties.	If the library is screened at high concentrations, low-potency effects could cloud the interpretation of the results.	Initial screen already informs about toxicity of compounds (organism viability). Hits already have “drug-like” properties.	If the library is screened at high concentrations, low-potency effects could cloud the interpretation of the results. However, if too low concentrations are used, then no effect may be seen because drug concentrations in the organism tend to be much smaller than those in the medium. Toxic compounds are eliminated even though they might have pharmacological properties and less toxic variants could possibly be made.
**Lead optimization**	Very amendable for lead optimization (SAR).			Exclusion of hits that have poor pharmacokinetic and pharmacodynamic properties but that could still be amendable to medicinal chemistry optimization. In addition, lead optimization (SAR) can be more difficult.		Exclusion of hits that have poor pharmacokinetic and pharmacodynamic properties but that could still be amendable to medicinal chemistry optimization. In addition, lead optimization (SAR) can be more difficult.
**Amount of compound required**	Low amounts of compound required.		Low amounts of compound required.			Large amounts of compound required.
**Throughput**	Very high throughput		High throughput			Low throughput

**Table 2:** A comparison of the various automated lifespan machines for *C. elegans.* NGM, nematode growth medium; FUdR, 5-fluoro-2-deoxyuridine.

	Manual	Wormbot	Automated Lifespan Machines	WorMotel	Microfluidics	LFASS
**Culture medium**	Liquid or NGM	NGM	Modified version of NGM	NGM	Liquid	See manual, assay in liquid
**FUdR**	Optional	Needed	Needed	Needed	Not needed	Generally needed
**Throughput**	Very low	High (144 wells)	Moderate (16 Petri plates)	High (240 wells)	Low (depends on used chip)	Very high (96- or 384-well plates)
**Temporal resolution**	Very low	High	High	High	High	Very high
**Individual/population**	Both are possible	Population	Population	Individual	Both are possible	Population
**Equipment cost (excluding labor cost)**	Very low	High	Moderate	Very high	Moderate	Moderate
**Automated data collection and analysis**	No	Yes	Yes	Yes	Depends	Yes
